# Myogenic differentiation of human myoblasts and Mesenchymal stromal cells under GDF11 on Poly-ɛ-caprolactone-collagen I-Polyethylene-nanofibers

**DOI:** 10.1186/s12860-023-00478-1

**Published:** 2023-05-15

**Authors:** Aijia Cai, Paul Schneider, Zeng-Ming Zheng, Justus P. Beier, Marcus Himmler, Dirk W. Schubert, Volker Weisbach, Raymund E. Horch, Andreas Arkudas

**Affiliations:** 1 Department of Plastic and Hand Surgery, Laboratory for Tissue Engineering and Regenerative Medicine, University Hospital of Erlangen, Friedrich-Alexander University of Erlangen-Nürnberg (FAU), 91054 Erlangen, Germany; 2 Department of Plastic Surgery, Hand Surgery - Burn Center, University Hospital RWTH Aachen, 52074 Aachen, Germany; 3 Institute of Polymer Materials, Department of Materials Science and Engineering, University of Erlangen-Nürnberg (FAU), 91058 Erlangen, Germany; 4 Department of Transfusion Medicine, University Hospital of Erlangen, Friedrich-Alexander- University Erlangen-Nürnberg (FAU), 91054 Erlangen, Germany

**Keywords:** Myogenic differentiation, Nanoscaffolds, Electrospun composite nanofibers, poly-ε-caprolacton (PCL), Collagen, Polyethylene oxide (PEO), ADSC, Myoblasts, GDF11, Myostatin

## Abstract

**Background:**

For the purpose of skeletal muscle engineering, primary myoblasts (Mb) and adipogenic mesenchymal stem cells (ADSC) can be co-cultured and myogenically differentiated. Electrospun composite nanofiber scaffolds represent suitable matrices for tissue engineering of skeletal muscle, combining both biocompatibility and stability Although growth differentiation factor 11 (GDF11) has been proposed as a rejuvenating circulating factor, restoring skeletal muscle function in aging mice, some studies have also described a harming effect of GDF11. Therefore, the aim of the study was to analyze the effect of GDF11 on co-cultures of Mb and ADSC on poly-ε-caprolactone (PCL)-collagen I-polyethylene oxide (PEO)-nanofibers.

**Results:**

Human Mb were co-cultured with ADSC two-dimensionally (2D) as monolayers or three-dimensionally (3D) on aligned PCL-collagen I-PEO-nanofibers. Differentiation media were either serum-free with or without GDF11, or serum containing as in a conventional differentiation medium. Cell viability was higher after conventional myogenic differentiation compared to serum-free and serum-free + GDF11 differentiation as was creatine kinase activity. Immunofluorescence staining showed myosine heavy chain expression in all groups after 28 days of differentiation without any clear evidence of more or less pronounced expression in either group. Gene expression of myosine heavy chain (*MYH2*) increased after serum-free + GDF11 stimulation compared to serum-free stimulation alone.

**Conclusions:**

This is the first study analyzing the effect of GDF11 on myogenic differentiation of Mb and ADSC co-cultures under serum-free conditions. The results of this study show that PCL-collagen I-PEO-nanofibers represent a suitable matrix for 3D myogenic differentiation of Mb and ADSC. In this context, GDF11 seems to promote myogenic differentiation of Mb and ADSC co-cultures compared to serum-free differentiation without any evidence of a harming effect.

**Supplementary Information:**

The online version contains supplementary material available at 10.1186/s12860-023-00478-1.

## Background

Muscle regeneration is orchestrated by muscle satellite cells, adult stem cells, located in a niche between the basal lamina and the sarcolemma. Those satellite cells from a pool of myogenically committed cells, the myoblasts, which fuse and differentiate into multinucleated myotubes and eventually into myofibers [1, 2]. In the event of volumetric muscle loss, the natural regeneration capacity of skeletal muscle tissue is exceeded [3]. To overcome the issue of donor site morbidity when using autologous muscle tissue to reconstruct the resulting defect, tissue engineering approaches for creating functional skeletal muscle tissue have been investigated thoroughly [3–6]. Since skeletal muscle represents a complex tissue with hierarchically organized fibers, a matrix, mimicking those properties is needed [7]. A prior study demonstrated that poly-ε-caprolacton (PCL)-collagen I nanofibers, electrospun with acetic acid combined with ultrasonic treatment as a benign solvent system, served as a suitable platform for those tissue engineering purposes due to their parallel alignment, stability, and biocompatibility [4, 8, 9]. However, high density packed fibers might result in poor cell infiltration [10]. One method to improve cell permeability into the scaffolds is to integrate polyethylene oxide (PEO) as sacrificial fibers to increase porosity of nanoscaffolds [11, 12].

In prior studies, Mb were co-cultured with mesenchymal stromal cells (MSC) for the purpose of tissue engineering means. Unlike Mb, MSC can be easily expanded without losing their differentiation ability [4, 9, 13]. In this context, MSC from bone marrow (BMSCs) or from adipose tissue (ADSC) were co-cultured with primary Mb three-dimensionally (3D) on PCL-collagen I-nanoscaffolds and myogenically differentiated. ADSC seemed superior in terms of proliferation and cell viability compared to BMSC [4]. To eliminate potential inconsistencies and safety concerns, a serum-free medium for myogenic differentiation was established. All in all, the establishment of this biocompatible system has been a crucial step in terms of future translational applications. However, gene expression of late myogenic markers like myosin heavy chain (MHC) was still higher after long term stimulation with donor horse serum (DHS) compared to serum-free differentiation [4]. Thus, the optimization of a serum-free medium for myogenic differentiation is crucial in terms of clinical translation of models of skeletal muscle tissue engineering.

Growth differentiation factor 11 (GDF11), a member of the transforming growth factor (TGF)-beta superfamily [14], has shown beneficial effects on skeletal muscle regeneration, restoring satellite cell regenerative function in aged muscle cells and mice [15] and was identified as a rejuvenation factor in heterochronic parabiosis experiments [16, 17]. It was suggested that GDF11 declines with age, and that restoration of systemic GDF11 to „youthful“ levels is beneficial for several age-related conditions [18]. Sinha et al. were able to show that supplementation of GDF11 reversed functional impairments and restored genomic integrity in aged muscle stem cells and mice, suggesting rejuvenating effects of GDF11 on aging skeletal muscle. The authors concluded that GDF11 acts both directly and indirectly to restore satellite cell regenerative function [15]. Contrary to those results, others showed that GDF11 inhibited muscle regeneration and decreased satellite cell expansion in mice [19] and that GDF11 exposure in mice induced whole body wasting and profound loss of function in cardiac and skeletal muscle [20]. All in all, conflicting results as to the effect of GDF11 on muscle regeneration exist.

In order to better understand the effect of GDF11 on myogenic differentiation, the aim of this study was to analyze its effect on ADSCs co-cultured with Mb on PCL-collagen I-PEO-nanofibers under serum-free conditions.

## Results

### Human myoblast and ADSC characterization

**Fig. 1:**
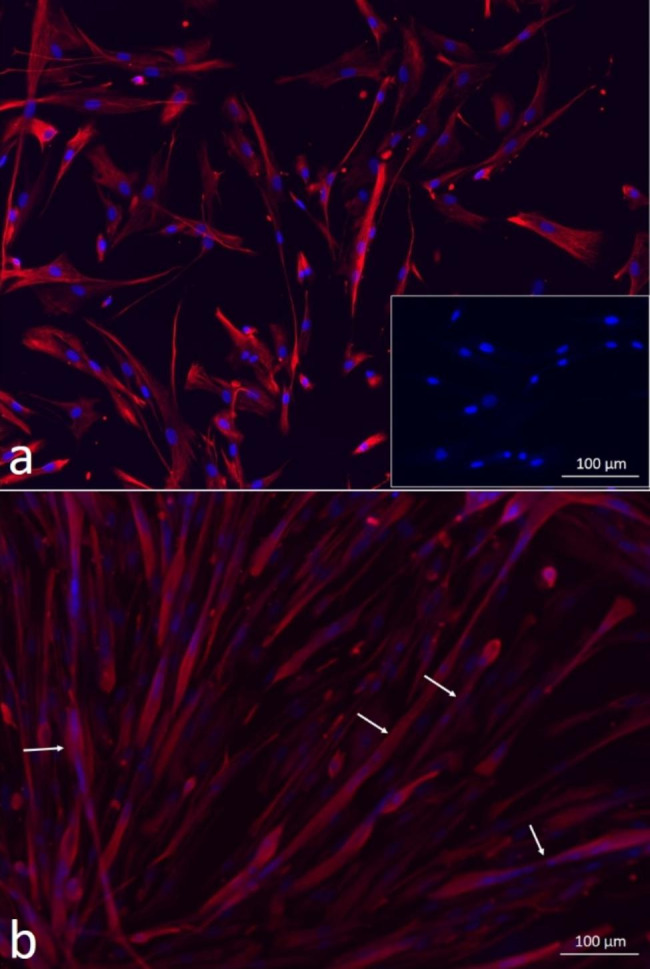
**(a)** Mb in P6 showed > 95% positive staining for the muscle-specific marker desmin. Human primary fibroblasts in P9 served as negative control (insert); **(b)** Mb in P6 showed formation of multinucleated myotubes (exemplarily marked with white arrows) after 7 days of myogenic differentiation

Human skeletal Mb in passage 6 (P6) showed > 95% positive staining for the muscle-specific marker desmin. After 7 days of myogenic differentiation, there was evidence of multinucleated myotube formation (Fig. 1a and b).

Extensive ADSC characterization has been performed in a prior study [21]. Briefly, they were successfully differentiated into the chondrogenic, osteogenic, and adipogenic lineage and showed positive expression for CD90, CD73, and CD105 while negative MSC markers CD34, CD11b, CD19, CD45, HLA-DR, CD31, CD271, and SSEA-4 (stage-specific embryonic antigen-4) were not expressed in both passage 3 (P3) and passage 6 (P6) in a subsequent FACS analysis [21]. This phenotype is characteristic for MSC [22].

### Optimal GDF11 concentration for myogenic differentiation of mb and ADSC co-cultures

**Fig. 2:**
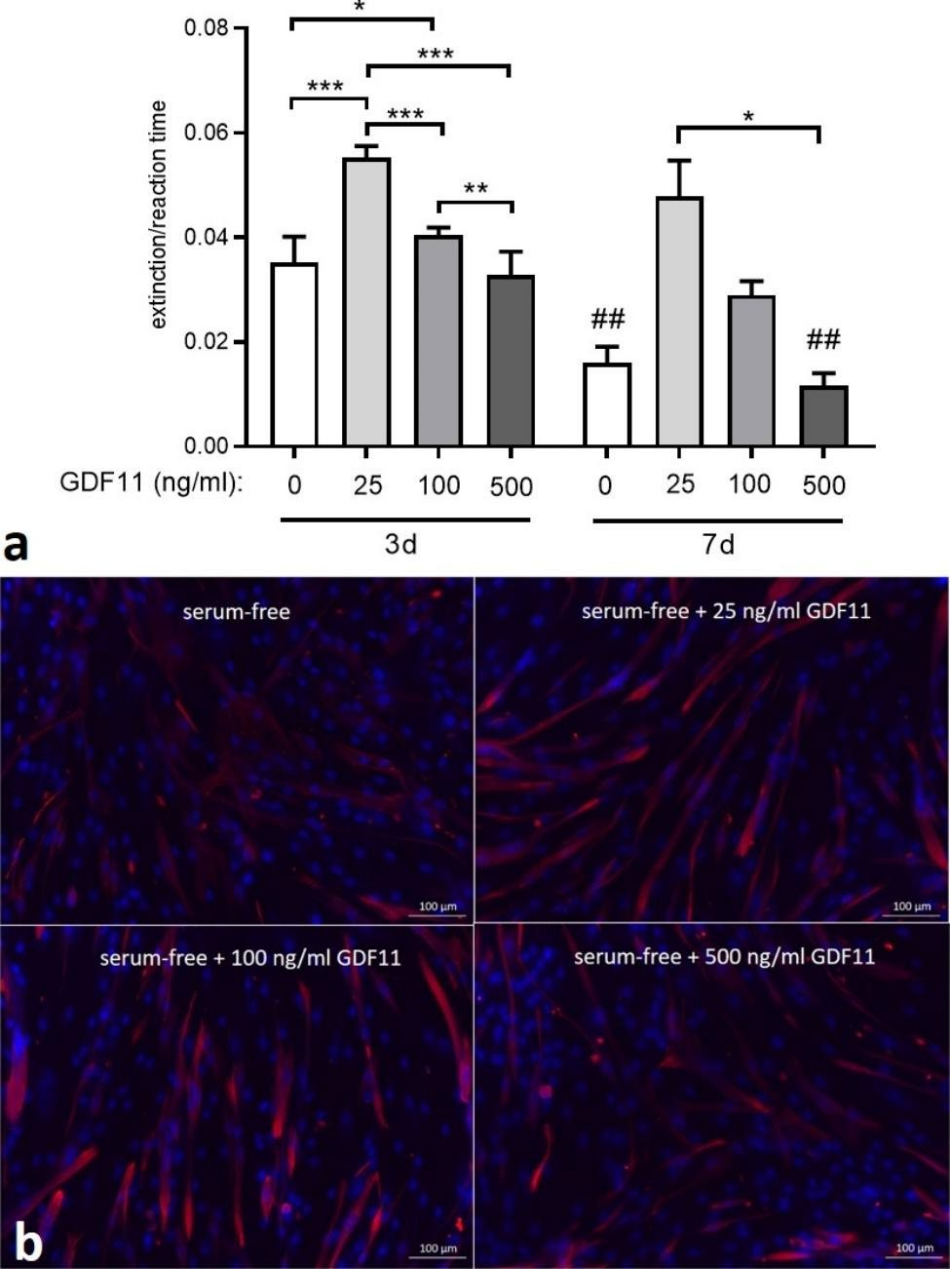
**(a)** CK activity after 3 and 7 days of myogenic differentiation of 2D co-cultures of Mb and ADSC under serum-free conditions (0 ng/ml GDF11). Multiple comparisons between different groups within the same time period were performed with one-way ANOVA with Tukey‘s multiple comparisons test or Friedman test with Dunn‘s correction as appropriate (n = 3). Levels of significance were * p < 0.05, ** p < 0.01, *** p < 0.001. Paired t-test was used to compare same groups over different time periods (3 days vs. 7 days) (n = 3). Level of significance was ## p < 0.01; **(b)** Desmin staining of co-cultures of Mb and ADSC after 7 days of differentiation under serum-free (top left), serum-free + 25 ng/ml GDF11 (top right), serum-free + 100 ng/ml GDF11 (bottom left), and serum-free + 500 ng/ml GDF11 (bottom right) differentiation

Co-cultures of Mb and ADSC were seeded as monolayers and stimulated with serum free differentiation medium supplemented with 3 different concentrations of GDF11: 25 ng/ml, 0.1 µg/ml, and 0.5 µg/ml. After 3 days of myogenic differentiation, there was decreasing creatine kinase (CK) activity with increasing GDF11 concentration (Fig. 2a). Statistical analysis showed significant differences between all groups with highly significant differences between 25 ng/ml GDF11 and all other GDF11 concentrations, including serum-free media without addition of GDF11 (p < 0.001). There was no difference between serum-free media and 500 ng/ml GDF11 (p = 0.4). After 7 days, pairwise comparisons showed a significant decrease of CK activity after serum-free differentiation (p = 0.0037) and with addition of 100 ng/ml GDF11 (p = 0.0071), but not with 25 ng/ml GDF11 (p = 0.11) and 500 ng/ml GDF11 (p = 0.25), although there was a tendency towards lower CK activity for both concentrations. Similarly, desmin staining after 7 days of myogenic differentiation showed formation of multinucleated myotubes under serum-free + 25 ng/ml GDF11 and serum-free + 100 ng/ml (Fig. 2b). Myotube fusion index (MFI) was highest for serum- free + 25 ng/ml GDF11 (0.159) while MFI was 0.078, 0.071, and 0.034 for serum-free + 100 ng/ml GDF11, serum-free alone, and serum-free + 500 ng/ml GDF11, respectively. Based on those results, a GDF11 concentration of 25 ng/ml was chosen for further experiments.

### Characterization of PCL-collagen I-PEO-nanoscaffolds

**Fig. 3:**
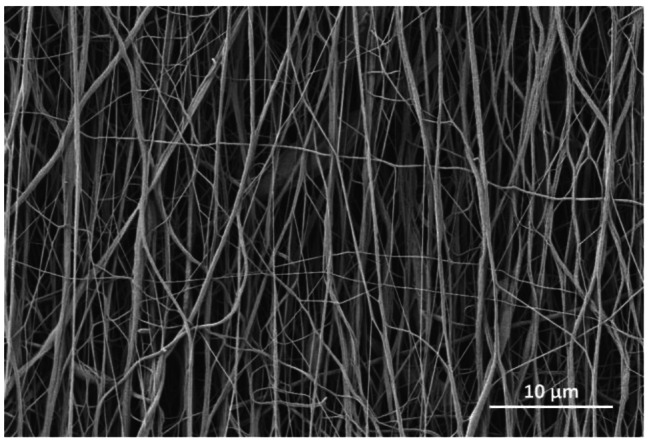
SEM image of aligned PCL-collagen I-PEO-nanofibers. Scale bar 10 μm

SEM images showed aligned PCL-collagen I-PEO-nanofibers (Fig. 3). Mean diameter of nanofibers was 233 ± 116 nm. Concerning fiber orientation, 68.27% of all fibers were within ± 4.6°, while 95.45% of all fibers were within ± 9.2° of main fiber orientation. Single-fiber tensile test revealed an Elastic Young’s modulus of 21.3 ± 7.3 MPa for PCL-collagen I-PEO-bundles.

### Cell viability on PCL-collagen I-PEO-nanoscaffolds

**Fig. 4:**
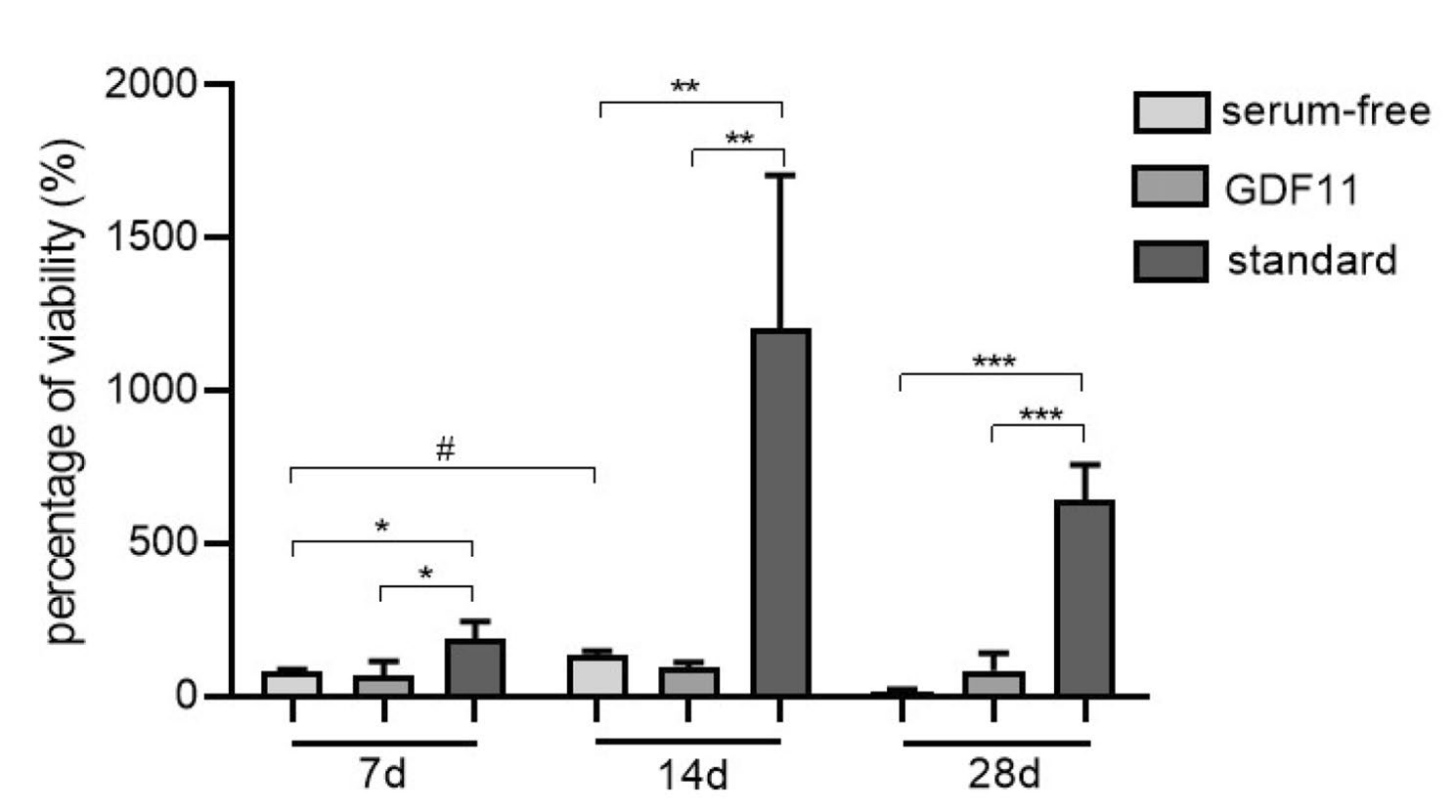
Wst-8-assay after 7, 14, and 28 days of myogenic differentiation of 3D co-cultures of Mb and ADSC on PCL-collagen I-PEO-nanofibers. Viability is shown as percentage of control (absorbance of cell-seeded scaffolds after 7 days of proliferation). Multiple comparisons between different groups within the same time period were performed with one-way ANOVA with Tukey‘s multiple comparisons test (n = 3). Levels of significance were * p < 0.05, ** p < 0.01, *** p < 0.001. Repeated measures ANOVA with Tukey‘s multiple comparisons test was used to compare same groups over different time periods (n = 3). Level of significance was # p < 0.05

Cell viability of 3D co-cultures of Mb and ADSC on PCL-collagen I-PEO-nanoscaffolds was assessed after 7, 14, and 28 days of myogenic differentiation. While there was no difference between group 1 (serum-free) and group 2 (serum-free + GDF-11) (p = 0.952), cell viability was higher in group 3 (standard) compared to group 1 (p = 0.048) and group 2 (p = 0.034). With increasing differentiation time, this difference became more pronounced: After 14 days, cell viability of group 3 increased highly significantly compared to group 1 (p = 0.010) and group 2 (p = 0.008). There was no difference between group 1 and 2 (p = 0.984). After 28 days, cell viability also differed between group 3 and group 1 in favor of group 3 (p = 0.001) and between group 3 and group 2 in favor of group 3 (p = 0.0003). Over time, cell viability did not increase within groups except within 7 to 14 days for group 1 (p = 0.030). There was a trend towards higher cell viability after 28 days compared to 7 days for group 3 (p = 0.080) (Fig. 4).

### Myogenic differentiation on PCL-collagen I-PEO-nanoscaffolds

**Fig. 5:**
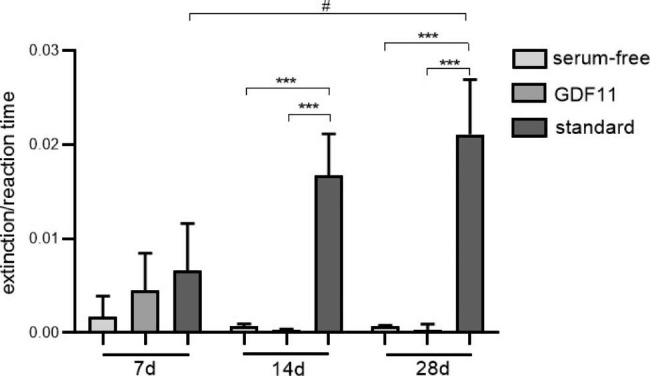
CK assay after 7, 14, and 28 days of myogenic differentiation of 3D co-cultures of Mb and ADSC on PCL-collagen I-PEO-nanofibers. Multiple comparisons between different groups within the same time period were performed with one-way ANOVA with Tukey‘s multiple comparisons test or Kruskal-Wallis test with Dunn‘s correction as appropriate (n = 3). Level of significance was *** p < 0.001. Repeated measures ANOVA with Tukey‘s multiple comparisons test was used to compare same groups over different time periods (n = 3). Level of significance was # p < 0.05

CK assay showed no difference between groups after 7 days of myogenic differentiation (p = 0.439). After 14 days and 28 days, CK activity highly significantly increased in group 3 (standard) compared to group 1 (serum-free) (p = 0.001) and group 2 (serum-free + GDF11) (p = 0.001). There was no difference between group 1 and group 2 during all time periods (p > 0.999 after 7 days, p = 0.986 after 14 days, p = 0.991 after 28 days). Over time, CK activity did not change within groups, except in group 3, where CK activity increased from 7 days to 28 days (p = 0.031) (Fig. 5).

**Fig. 6:**
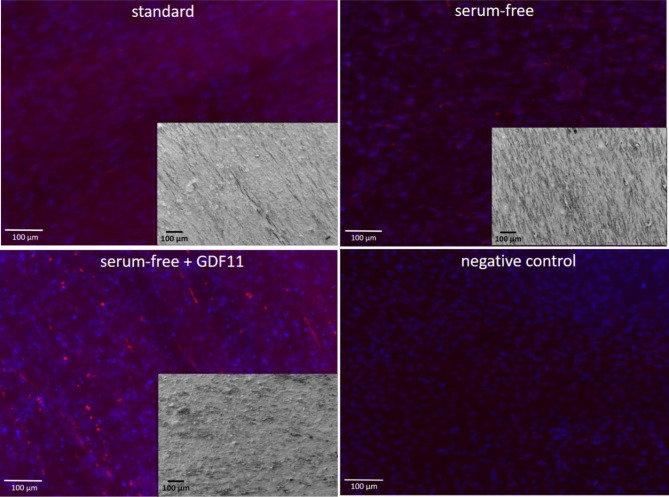
Myosin heavy chain (MHC) immunofluorescent staining (red = MHC, blue = DAPI) of co-cultures of Mb and ADSC on PCL-collagen I-PEO-nanofibers. Co-cultures were myogenically differentiated for 28 days in serum-free media (up right), serum-free media + GDF11 (down left), or standard serum-containing media (up left). Fibroblasts were allowed to proliferate on nanofibers for 1 week and served as negative control (down right). Scanning electron microscopy (SEM), showing configuration of attached cells on PCL-collagen I-PEO-nanofibers are represented as inserts in the corresponding fluorescent microscopy image

With scanning electron microscopy (SEM) images, configuration of attached cells on PCL-collagen I-PEO-nanoscaffolds could be analyzed (Fig. 6, Supplementary Figures S1-3). After 28 days of myogenic differentiation, co-cultures of Mb and ADSCs showed parallel alignment on the scaffolds under all three differentiation conditions. While in group 2 (serum-free medium + 25 ng/ml GDF11) and group 3 (standard), cells covered almost the entire surface of the scaffold, the cells in group 1 (serum-free) were not as densely packed. Fluorescence microscopy showed a similar trend after 28 days of serum free, serum free + GDF11, and standard myogenic differentiation. All groups led to positive expression of myosin heavy chain (MHC) of differentiated Mb and ADSC co-cultures compared to negative control (Fig. 6). Fluorescent intensity ratio of MHC positive cells to diamidine-phenylindole-dihydrochloride (DAPI) was 0.989 for group 1, 1.045 for groups 2, and 0.957 for group 3.

**Fig. 7:**
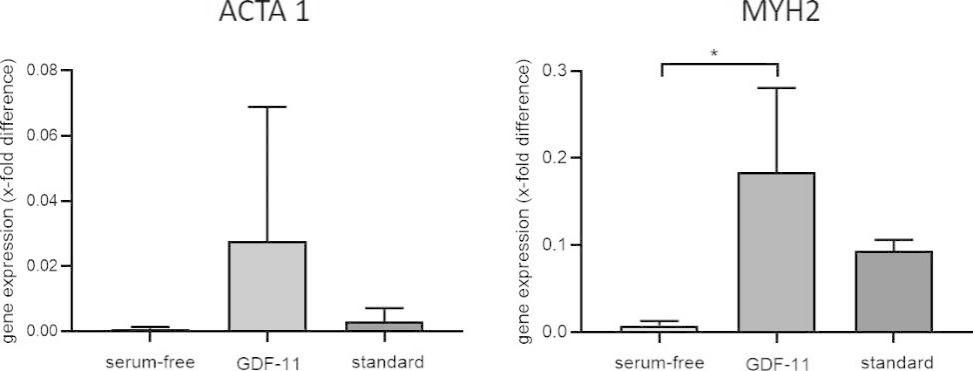
Gene expression of the late myogenic markers myosine heavy chain (*MYH2*) and actin alpha skeletal muscle (*ACTA1*) in co-cultures of Mb and ADSC after 28 days of myogenic differentiation. Expressions are demonstrated in x-fold difference compared with RNA from human muscle tissue using the 2^−ΔΔCt^ method. *GAPDH* was used as housekeeping gene in co-cultures and muscle tissue. Comparisons between groups were performed with one-way ANOVA with Tukey‘s multiple comparisons test or Kruskal-Wallis test with Dunn‘s correction as appropriate (n = 3). Level of significance was * p < 0.05

Gene expression of the skeletal muscle motor protein MYH2 (myosin heavy chain 2) increased after 28 days of serum-free differentiation with GDF11 (group 2) compared to serum-free differentiation alone (group 1) (p = 0.021) while there was neither a difference between group 1 and group 3 (standard) (p = 0.229) nor between group 2 and group 3 (p = 0.206). There was no change in the cytoskeletal protein ACTA1 (skeletal alpha actin) expression between either group (group 1 vs. group 2: p = 0.303, group 1 vs. group 3: 0.890, group 2 vs. group 3: p = 1.000) (Fig. 7).

## Discussion

The findings of this study implicate that Mb and ADSC co-cultures showed adequate viability and adherence on PCL-collagen I-PEO-nanofibers. Myogenic differentiation over a period of 28 days led to the expression of myogenic key markers and parallel arrangement along the aligned nanofibers. A low concentration of 25 ng/ml GDF11 led to positive MHC expression as shown by immunostaining and enhanced MYH2 gene expression of Mb and ADSC co-cultures on PCL-collagen I-PEO-nanoscaffolds at least under serum-free differentiation conditions. MYH2 and ACTA1 are both terminal myogenic markers guiding sarcomere formation [9]. On the other hand, GDF11 was not beneficial in terms of cell viability and myogenic differentiation, assessed by CK activity and even inhibited those parameters when compared to standard serum-containing differentiation.

The role GDF11 has on skeletal muscle regeneration is controversial as both beneficial as well as harming effects have been described [19, 20, 23]. This might be explained by GDF11 being closely related to GDF8 or myostatin, which is a potent inhibitor of skeletal muscle growth [24]. Both myostatin and GDF11 act via the activin type II receptor, activating the Smad2-/Smad3-complex, and thereby mediating downstream signaling [25, 26]. The effect of GDF11 on MSC has also been studied intensely. For instance, GDF11 inhibited MSC to differentiate into the adipogenic lineage via the TGF-beta signaling pathway [27]. GDF11 also inhibited osteoblast and chondrogenic differentiation of bone marrow-derived MSC [28, 29]. The influence of GDF11 on myogenic differentiation capacity of MSC let alone co-cultures of primary Mb and ADSC on PCL-collagen I-PEO-nanoscaffolds has not been investigated so far.

The results of the present study reflect a similar trend, but they do not clearly support the previously mentioned studies. CK activity, which has been used as a biochemical marker for direct measurement of myogenic differentiation [20, 30], decreased over time after induction of serum-free myogenic differentiation, which is in accordance to a previous study [4]. As described in the aforementioned study, decrease in CK activity could have been evoked by a decline in growth factors by using serum-free medium [4]. Additional GDF11 did not lead to an increase in CK activity in the present study. When using GDF11 at a high concentration of 500 ng/ml, CK activity even decreased over time, which could be explained by an inhibiting effect of GDF11 on myogenic differentiation. More recent findings support this theory and demonstrate that GDF11 is not the long-sought rejuvenating factor as opposed to the theory of Sinha et al. [15, 31]. Egerman et al. showed that previously used reagents to detect GDF11 were nonspecific and that GDF11 rather increases with age and inhibits muscle regeneration and myoblast differentiation [19].

On the other hand, no difference in GDF11 concentration could be detected via enzyme-linked immunosorbent assay between plasma of younger compared to older individuals [32]. In a recent study, low concentrations of 1 ng/ml and 10 ng/ml of GDF11 have not led to a decrease in myogenic differentiation but rather to an increase in nuclear density and myotube length and width of different myogenic cells [33]. Similarly, in the present study, the addition of GDF11 did not lead to a decrease of CK activity and even upregulated MYH2 compared to serum-free differentiation alone. However, both groups were still not as stimulating as the standard serum-containing differentiation medium. As described in a previous study, myogenic differentiation of MSC necessitates growth factors, which might not be contained in Ultroser® G [4]. Other growth factors besides GDF11 might be necessary for an increase of myogenic differentiation to the level of stimulation with serum-containing differentiation medium, for example insulin-like growth factor-1 [9, 34] or human epidermal growth factor [35]. At least, GDF11 did not lead to a significant decrease of myogenic key markers as opposed to the findings by Egerman et al. [19]. The effect of GDF11 in combination with the standard serum-containing medium remains unclear as this combination was not tested in the present study since the main goal was to test the effect of GDF11 independent of potential growth factors of unknown composition as contained in serum.

Electrospun PCL-collagen I-nanoscaffolds have proven to be a suitable and biocompatible matrix for long-term differentiation of co-cultures of Mb and ADSC [4]. The integration of PEO sacrificial fibers is supposed to increase porosity of nanoscaffolds, facilitating cell influx [11, 12]. Unfortunately, we did not compare the newly established PCL-collagen I-PEO-nanoscaffolds to PCL-collagen I-nanoscaffolds. Thus, conclusions about a more efficient myogenic induction of either of those blended fiber types cannot be made. Yet, we were able to demonstrate positive MHC expression in this setting of 28 days of myogenic differentiation. A prior analysis of 3D vascularization of PCL-collagen I-nanofiber scaffolds *versus* PCL-collagen I-PEO-nanofiber scaffolds in the arteriovenous loop model of the rat showed better integration into the host organism for the PEO-blended fibers [12]. However, the PCL-collagen I-PEO-nanofibers in that study were randomly arranged. A more recent study, which had been completed simultaneously to the current study, compared aligned PCL-collagen I-nanofiber scaffolds to aligned PCL-collagen I-PEO-nanofiber scaffolds, which were similar to the scaffolds used in the current study, in an in vivo vessel loop model. Surprisingly, the mentioned study revealed higher amount of vascularization and angiogenesis for PCL-collagen I-nanofibers compared to PCL-collagen I-PEO-nanofibers. The authors concluded that pores could have led to a certain degree of loss of mechanical stability of PCL-collagen I-PEO-nanofibers compared to PCL-collagen I-nanofibers, fostering tissue ingrowth [36]. Thus, further comparison between those two scaffolds types is a prerequisite for future studies before this 3D model can be translated into clinical applications.

There are several limitations of this study. First, standard deviations were sometimes high which might be explained by the limited sample size and the use of primary human cells of different subjects. The purchased myoblasts were all from the quadriceps muscle and human subjects were all male to limit heterogeneity. Other factors like age are known to influence the degree of myotube formation [37, 38]. Furthermore, maintenance and myogenic differentiation of primary cells isolated from adult muscle tissue is a larger challenge than culturing immortal cell lines like C2C12 with a rapid proliferative and differentiation capacity [39]. However, cell lines are not suitable for establishing a model of skeletal muscle engineering, which can be translated into clinical application since they do not represent the characteristics of skeletal muscle as accurately as primary myoblasts do [39]. Second, the 3D structure of the cell-seeded nanoscaffolds impeded taking clear immunofluorescent pictures with a fluorescence microscope. Configuration of attached cells could only be detected via SEM, but myotube formation could not be seen by this method. This could also be ascribed to the properties of the PCL-collagen I-PEO_nanofibers for the reasons described above. Nevertheless, confocal microcopy should be used for future studies involving cell-seeded 3D nanoscaffolds [39]. After all, a clear difference could be shown between probes and negative control. Mb monocultures were not analyzed, so it is uncertain what effect GDF11 has on Mb in our experimental setting. On the other hand, Mb alone might not be promising for engineering large-scale tissue, which can eventually be applied in the patient since they have a limited proliferation capacity [7, 40, 41]. In several studies, Mb and ADSC co-cultures were differentiated into the myogenic lineage [4, 42, 43]. Gehmert et al. were able to show that ADSC secreted IGF-1 that protected myoblasts from negative effects of myostatin [4, 42, 43]. After exposure to ADSC-conditioned medium, myostatin treated myoblasts showed less apoptosis and more proliferation as well as higher expression of MyoD [4, 42, 43], a marker of myogenic commitment [4, 42, 43]. Oki et al. found similar results and discovered that decorin, a dermatan sulfate proteoglycan and known inhibitor of TGFbeta1, was secreted by ADSCs, protecting myoblasts against fibrosis [4, 42, 43]. The protective effects of ADSC might have competed with the supposedly negative effects of GDF11, which led to the presented ambiguous effects of GDF11 on Mb and ADSC co-cultures. Another benefit of ADSC is their relatively easy and safe harvesting method and their high expansion capacity, making them an attractive cell source for co-culture with primary myoblasts [4, 42, 43]. In the current study as well as in prior studies, ADSC in higher passage (P6) displayed the same characteristics as ADSC in lower passage (P3) [4] [21]. Thus, ADSC in P6 were used for experiments. This has an impact on later clinical translation since a small amount of harvested tissue results in abundant number of cells. Specifically in cases of muscular diseases as Duchenne muscular dystrophy where myoblasts cannot be isolated due to depletion of the satellite cell pool ADSC would be the sole cell source for skeletal muscle tissue engineering purposes [44]. Thus, an ADSC monoculture system is of particular interest for future studies.

Given the results of the present study, we propose that PCL-collagen I-PEO-nanofibers are a viable option for myogenic differentiation of Mb and ADSC co-cultures. In this setting, GDF11 alone might be promoting myogenic differentiation of Mb and ADSC co-cultures under serum-free conditions. We were not able to prove a harming effect of the myostatin homologue as proposed by recent studies [4, 42, 43]. A combination of GDF11 with other proteins, particularly those secreted by ADSC should be analyzed in terms of the effect on myogenesis under serum-free conditions as serum-free + GDF11 stimulation was not beneficial on cell viability compared to standard serum-containing myogenic differentiation. Regardless of the effects of GDF11, this biocompatible 3D model, consisting of primary Mb and ADSC co-cultured on PCL-collagen I-PEO-nanofibers can serve as a platform for skeletal muscle tissue engineering, which can be transferred to further in vivo and translational research. Other nanofiber scaffolds like PCL-collagen I-nanofibers are also viable options and should be directly compared to PCL-collagen I-PEO-nanofibers in terms of myogenic differentiation of Mb and ADSC.

## Conclusion

We have established electrospun aligned PCL-collagen I-PEO-nanofiber scaffolds as a novel biocompatible matrix for skeletal muscle tissue engineering purposes. Mb and ADSC showed adequate cell adherence, viability, and myogenic differentiation when co-cultured on those matrices. In this setting of serum-free myogenic differentiation, GDF11 had a promoting effect on myogenic differentiation of Mb and ADSC co-cultures, although this serum-free differentiation system was still inferior to serum-containing differentiation.

## Methods

### Human cell culture and characterization

Human Mb were purchased from Lonza (Walkersville, MD) and cultured in SkBM^TM^-2 Basal Medium with supplements (catalog #CC-3246 and #CC-3244, all from Lonza). Cells from quadriceps muscle from three different adult male donors (catalog #CC-2580, HSMM 33,406/Lot# 650,386, HSMM 30,551/Lot# 583,849, HSMM 33,607/Lot# 655,307) were passaged up to P6. Desmin immunofluorescence (ab8470, Abcam, Cambridge, UK) as described in detail under “Immunofluorescence” was used to confirm myogenic characteristics of the Mb. Human primary fibroblasts (HFIB-D, cryo, provitro AG, Berlin, Germany) in passage 9 (P9) served as negative control. To verify myotube formation capacity, Mb of P6 were stained for desmin after 7 days of myogenic differentiation induced by myogenic differentiation medium, containing DMEM/Ham’s F12 + 2% donor horse serum (DHS) + 1% L-Glutamin + 1% Penicillin/Streptomycin (P/S) (all from Biochrom GmbH, Berlin, Germany) + 0,4 µg/ml dexamethasone (Sigma Aldrich, St. Louis, Missouri, USA), 1 ng/ml basic fibroblast growth factor (bFGF) (Peprotech, Hamburg, Germany).

ADSC were isolated from human adipose tissue and characterized in P3 and in P6 as described previously [21]. Tissue collection was approved by the local Ethics Committee (approval number 424_18 B) in accordance with the World Medical Association Declaration of Helsinki and informed consent was obtained.

For each experiment, myoblasts from the three different donors were co-cultured with the ADSC in P6 at a ratio of 1:1 (n = 3). Since ADSC in P6 displayed the same characteristics as ADSC in P3 the higher passage of the both was used.

### Determination of optimal GDF11 concentration

Co-cultures of Mb and ADSC (n = 3) were seeded as monolayers in a ratio of 1:1 in 6-well culture plates at a density of 3 × 10^5^ cells in expansion medium containing DMEM/Ham’s F 12, 10% FCS, 1% L-Glutamin, 1% P/S (all from Biochrom GmbH). After 48 h, medium was replaced by serum free differentiation medium, containing DMEM/Ham’s F12 + 0.2% Ultroser® G (Cytogen GmbH, Wetzlar, Germany) as previously described [4]. Three different concentrations of GDF11 (LifeSpan BioSciences, Seattle, WA) were added to the serum free differentiation medium: 25 ng/ml, 0.1 µg/ml, and 0.5 µg/ml. The choice for the different concentrations was based on concentrations used for in vitro experiments reported in the literature [15, 19, 45]. Medium including fresh GDF11 was changed every 2–3 days. After 3 and 7 days, CK activity was colorimetrically determined (Abcam) as previously described [4]. The amount of nicotinamide adenine dinucleotide (NADH) generated by CK was determined photometrically at 450 nm with Thermo Scientific ™ Multiskan™ GO during minute 16–20 of reaction time since after 20 min, the activity of the samples was found to have reached a plateau. For further experiments, GDF11 in a concentration of 25 ng/ml was used. To visualize myogenic potential of GDF11 in the chosen concentration, Mb and ADSC were differentiated with serum free differentiation medium + 25 ng/ml GDF11 for 7 days and immunostained for desmin as described in detail under „Immunofluorescence“. Myotube fusion index (MFI) was calculated semi-automatically via ImageJ 1.53e (National Institutes of Health, Bethesda, MD, USA) as described previously [21].

### Electrospinning of PCL-collagen I-PEO-nanofibers and 3D cell culture

PCL-collagen I-PEO-nanofibers were produced by electrospinning [36]. Briefly, PCL (80.000 g/mol, Sigma Aldrich) was blended with bovine collagen type I (Symatese, Lyon, France) in a ratio of 2:1 at a 12% (w/v) solution, using acetic acid (90% v/v in dH_2_O, Carl Roth GmbH, Karlsruhe, Germany) as solvent. Parallel PCL-collagen I-nanofibers were electrospun on a standard electrospinning machine onto parallel metal rods on a custom-made rotating drum (15 kV, 15 cm, 1 ml/h, 50 rpm). PEO (concentration 10% (w/v), molecular weight: 900.000 g/mol, Sigma Aldrich) nanofibers were similarly spun (14 kV, 13 cm, 1 ml/h, 50 rpm). The single-fiber tensile test was performed on 5 different PCL-collagen I-PEO-fiber bundles as described by Munawar and Schubert [46]. The aligned PCL-collagen I and PEO-fibers were collected in alternate layers on plastic rings with 10 mm diameter (Minusheet carrier, Minucells and Minutissue Vertriebs GmbH, Bad Abbach, Germany). The area of the resulting scaffolds measured approximately 0,8 cm^2^. Scanning electron microscopy (SEM) images were taken as described below (“scanning electron microscopy”) and used for characterization of unseeded scaffolds. Fiber diameter was measured using ImageJ (National Institutes of Health, Bethesda, MD, USA, Version 1.53e). Fiber orientation was analyzed using the OrientationJ plugin (ImageJ) on 5 different images of the scaffolds taken in 10,000x magnification. This resulted in an orientation distribution histogram for each image. For cell seeding, scaffolds were sterilized in 70% ethanol, washed with PBS afterwards [36] and placed into 24 well-plates while they were soaked in DMEM/Ham’s F12 for approximately 1 h at 37 °C. ADSC and Mb were seeded with 100 µL thickened medium containing expansion medium and dissolved methyl cellulose (Sigma Aldrich) on PCL-collagen I-PEO-nanoscaffolds at 3 × 10^5^ cells in a ratio of 1:1. After 7 days of proliferation in expansion medium, differentiation was induced and continued for 28 days. As negative control for immunofluorescence staining, fibroblasts in P9 were seeded onto the nanoscaffolds as described above and allowed to proliferate in DMEM + 10% fetal calf serum (FCS) + 1% P/S (all from Biochrom GmbH) for 7 days.

### Myogenic differentiation conditions

To confirm myogenic differentiation potential of Mb in P6, monolayers were seeded in 48-wells at a cell density of 20.000 and allowed to proliferate in expansion medium for 2 days until cells reached confluence. Afterwards, differentiation was induced by switching to standard differentiation medium, containing 2% donor horse serum (DHS, Biochrom GmbH) (Table 1). Co-cultures of ADSC and Mb were seeded similarly into 48-wells and were allowed to differentiate with serum free differentiation medium + 25 ng/ml GDF11 (Table 1) for 7 days. Differentiation was continued for 7 days prior to desmin staining as described in detail under “Immunofluorescence”.

For three-dimensional (3D) co-cultures, ADSC and Mb were seeded onto PCL-collagen I-PEO-scaffolds and allowed to proliferate for 7 days as described above. After proliferation, co-cultures were myogenically differentiated under three different conditions: (1) DMEM/Ham’s F12 + 0.2% Ultroser® G + 1% L-Glutamin + 1% P/S + 0.4 µg/ml dexamethason + 1 ng/ml bFGF (Peprotech, Hamburg, Germany) (serum-free medium), (2) serum-free medium + 25 ng/ml GDF11, (3) DMEM/Ham’s F12 + 2% DHS + 1% L-Glutamin + 1% P/S + 0.4 µg/ml dexamethason + 1 ng/ml bFGF (standard differentiation medium, containing serum). For every experiment, 3 scaffolds per group were analyzed (n = 3). Mb from one donor (out of 3 in total) were seeded in co-culture with the ADSC onto one scaffold per group.

**Table 1:** Myogenic differentiation media

Group	Contains DMEM/Ham’s F12 + 1% L-Glutamin + 1% P/S + 0.4 µg/ml dexamethason + 1 ng/ml bFGF +
1) serum-free	0.2% Ultroser® G
2) serum-free + GDF11	0.2% Ultroser® G + 25 ng/ml GDF11
3) standard	2% DHS

### Cell viability and creatine kinase activity on PCL-collagen I-PEO-nanoscaffolds

3D co-cultures (n = 3) were allowed to proliferate for 7 days and subsequently to myogenically differentiate for 7, 14, and 28 days. After each time period, water-soluble tetrazolium salt (wst)-8-assay (Promokine, Promocell GmbH, Heidelberg, Germany) of the seeded scaffolds was performed as described previously [4]. Absorbance was measured at 450 nm with Photometer Thermo Scientific™ Multiskan™ GO to assess cell viability. Percentage of viability was shown after normalization of absorbance of each differentiation period to control (proliferation only). Afterwards, CK activity was determined as described above (n = 3).

### RNA isolation and quantitative PCR analysis

In 3D co-cultures (n = 3), the gene expression rate of the late myogenic markers *MYH2* (myosin heavy chain 2) and *ACTA1* (skeletal alpha actin) was analyzed as previously described [4]. As housekeeping gene, *GAPDH* (glyceraldehyde-3-phosphate dehydrogenase) was used as internal control. RNA of the samples was extracted using Trizol (Life Technologies, Carlsbad, CA, USA) and chloroform [21]. RNA was reverse-transcribed into cDNA using a QuantiTect Reverse Transcription Kit and a Sensiscript Reverse Transcription Kit (both from Qiagen GmbH). cDNA was amplified through quantitative real-time PCR using SsoAdvanced Universal SYBR Green PCR Supermix (Bio-Rad, Hercules, CA, USA) and Light Cycler (Bio-Rad CFX96 Touch™). Evaluation of gene expression was performed using the 2^−ΔΔCt^ method. RNA from human muscle tissue served as control sample. The primer sequences used are given in Table 2.

**Table 2:** Primer sequences

	Forward primer	Reverse primer
*MYH2*	GGGCCTTTCAAGAGGGACAC	TGCGCTCCCTTTCAGACTTT
*ACTA1*	CACAATGTGCGACGAAGACG	CTCTCTTGCTCTGAGCCTCG
*GAPDH*	TCCACCCATGGCAAATTCCA	TTCCCGTTCTCAGCCTTGAC

### Immunofluorescence

Mb in P6, myogenically differentiated Mb in P6, as well as differentiated co-cultures of ADSC and Mb were desmin-stained as previously described [4]. Briefly, cells were fixed with formaldehyde (Carl Roth GmbH), washed, and blocked in PBS with 1.5% FCS and 0.25% TritonX (Carl Roth GmbH) for one hour at room temperature. Cells were incubated with desmin primary antibody (ab8470, Abcam) at 0.5 µg/ml for one hour at room temperature.

ADSC and Mb were 3D co-cultured on PCL-collagen I-PEO-nanoscaffolds (n = 3) for 7 days before expansion medium was switched to differentiation medium (serum-free, serum-free + GDF11, standard). After 4 weeks, scaffolds were fixed, washed, blocked, and stained with anti-fast myosin skeletal heavy chain (MHC) antibody (ab91506, Abcam) at 5 µg/ml for one hour at room temperature.

Alexa Fluor 594 goat anti-mouse IgG1 cross-adsorbed secondary antibody (A-21,125, Thermofisher Scientific Inc.) was used as secondary antibody at 4 µg/ml for 30 min at room temperature for desmin stained cells and Alexa fluor 594 goat anti-rabbit IgG (H + L) (Thermofisher Scientific Inc.) was used as secondary antibody for MHC stained cells at the same conditions. After counterstaining with DAPI 1 µg/ml (Thermofisher Scientific Inc.) for 5 min, cells were subsequently analyzed and digitally photographed with a fluorescence microscope (IX83, cellSens, software, Olympus, Hamburg, Germany).

Human primary fibroblasts in P9 served as negative control.

Fluorescent intensity of MHC stained seeded nanoscaffolds was determined as a ratio of mean grey area of inverted blue channel and mean grey area of inverted red channel via ImageJ 1.53e (National Institutes of Health, Bethesda, MD, USA).

### Scanning electron microscopy

After immunofluorescence analysis of the seeded scaffolds after 28 days of myogenic differentiation, microstructural analysis of the scaffolds was performed using an Auriga Fib-scanning electron microscope (SEM) (Zeiss, Oberkochen, Germany) as described previously [4]. Probes were sputter-coated with gold using a Q150T Turbo-pumped Sputter Coater (Quorum Technologies Inc., Guelph, Canada).

### Statistical analysis

Shapiro-Wilk test was used to test data normality. Statistical analysis was performed with one-way analysis of variance (ANOVA) with Tukey’s multiple comparisons test or Kruskal-Wallis test with Dunn’s correction for multiple comparisons, as appropriate. Repeated measures ANOVA with Tukey’s multiple comparisons test was used for comparisons between matched variables at different time points for normally distributed data, otherwise Friedman test with Dunn’s correction for multiple comparisons was used. Pairwise comparison was done using paired t-test or Mann-Whitney test, as appropriate. Statistical analysis was performed using GraphPad Prism version 8.3 (La Jolla, CA, USA). A p-value ≤ 0.05 was considered statistically significant.

## Electronic supplementary material

Below is the link to the electronic supplementary material.

**Additional file 1** Scanning electron microscopy (SEM) of Mb and ADSC co-cultures on PCL-collagen I-PEO-nanofibers after 4 weeks of myogenic differentiation in standard serum-containing medium

**Additional file 2** Scanning electron microscopy (SEM) of Mb and ADSC co-cultures on PCL-collagen I-PEO-nanofibers after 4 weeks of myogenic differentiation in serum-free medium

**Additional file 3** Scanning electron microscopy (SEM) of Mb and ADSC co-cultures on PCL-collagen I-PEO-nanofibers after 4 weeks of myogenic differentiation in serum-free medium + 25 ng/ml GDF11

**Additional file 4**

## Data Availability

The datasets used and/or analysed during the current study are available from the corresponding author on reasonable request, qPCR-data are provided as supplementary file.
