# Supplementary figures and images for: Myogenic differentiation of human myoblasts and Mesenchymal stromal cells under GDF11 on Poly-ɛ-caprolactone-collagen I-Polyethylene-nanofibers
Source: BMC Mol Cell Biol. 2023 May 15;24:18. doi: 10.1186/s12860-023-00478-1 (PMC10184409; doi:10.1186/s12860-023-00478-1)

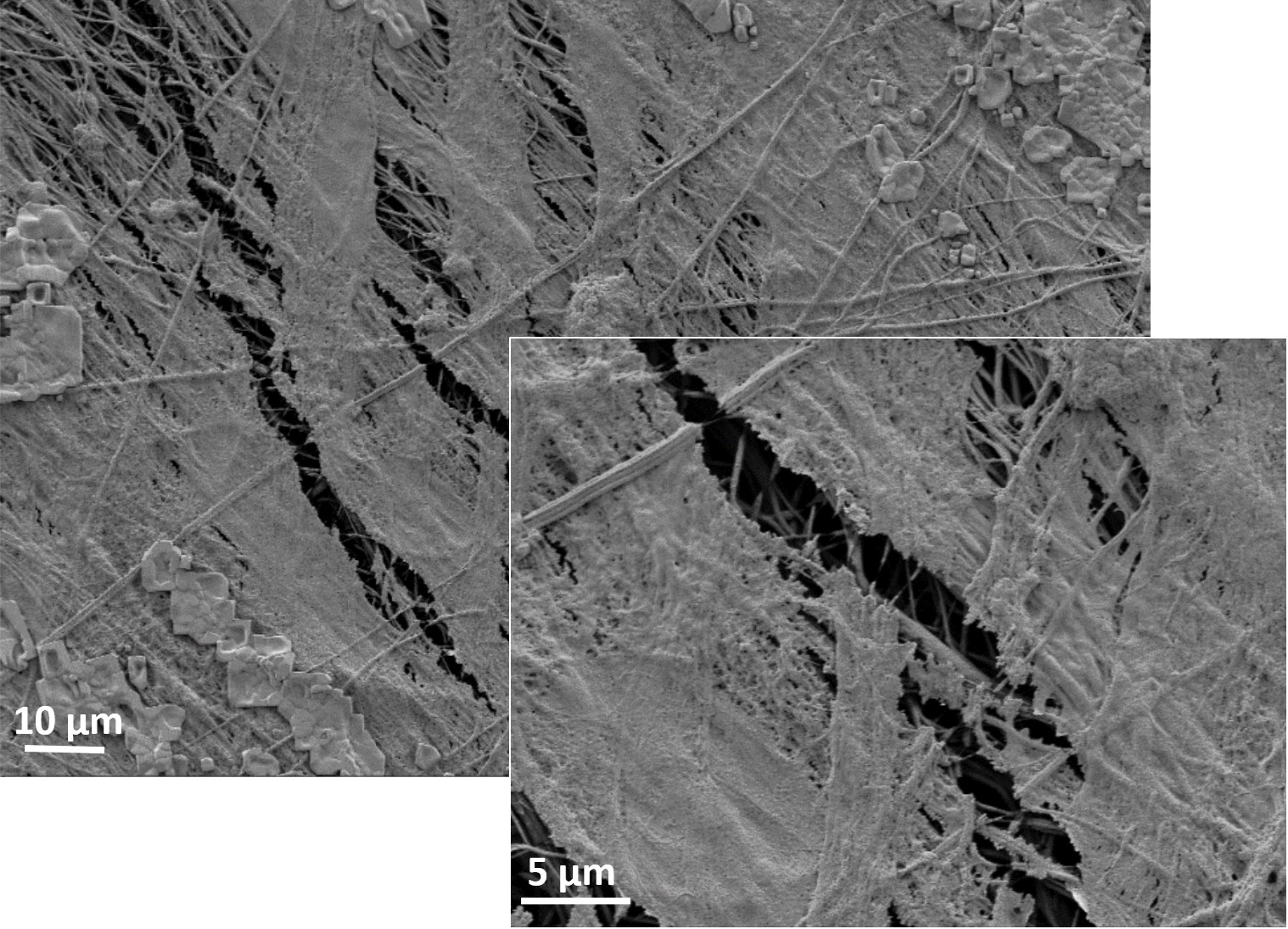

Supplement: Supplementary file 1 — Additional file 1 Scanning electron microscopy (SEM) of Mb and ADSC co-cultures on PCL-collagen I-PEO-nanofibers after 4 weeks of myogenic differentiation in standard serum-containing medium [file 12860_2023_478_MOESM1_ESM.tif]

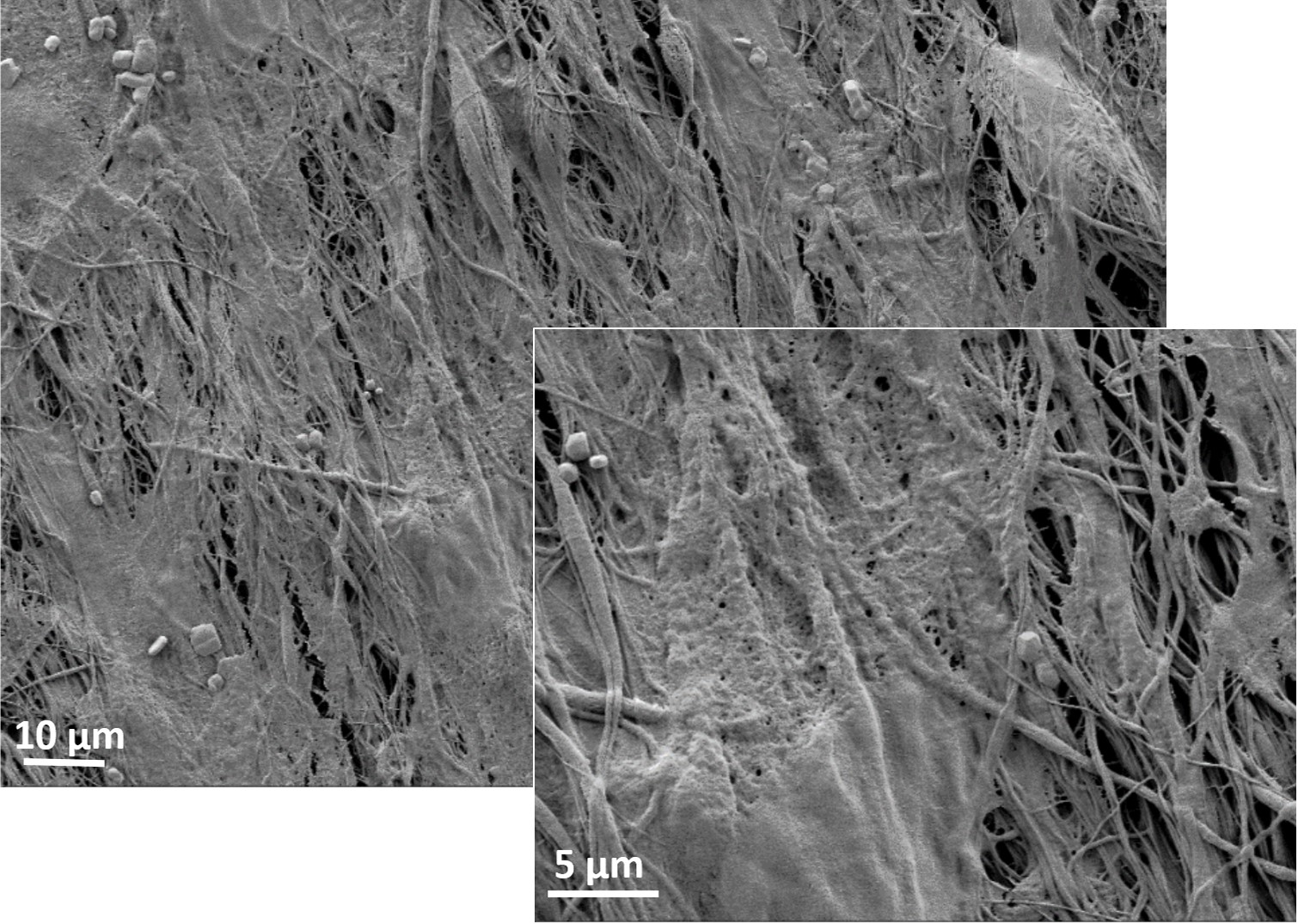

Supplement: Supplementary file 2 — Additional file 2 Scanning electron microscopy (SEM) of Mb and ADSC co-cultures on PCL-collagen I-PEO-nanofibers after 4 weeks of myogenic differentiation in serum-free medium [file 12860_2023_478_MOESM2_ESM.tif]

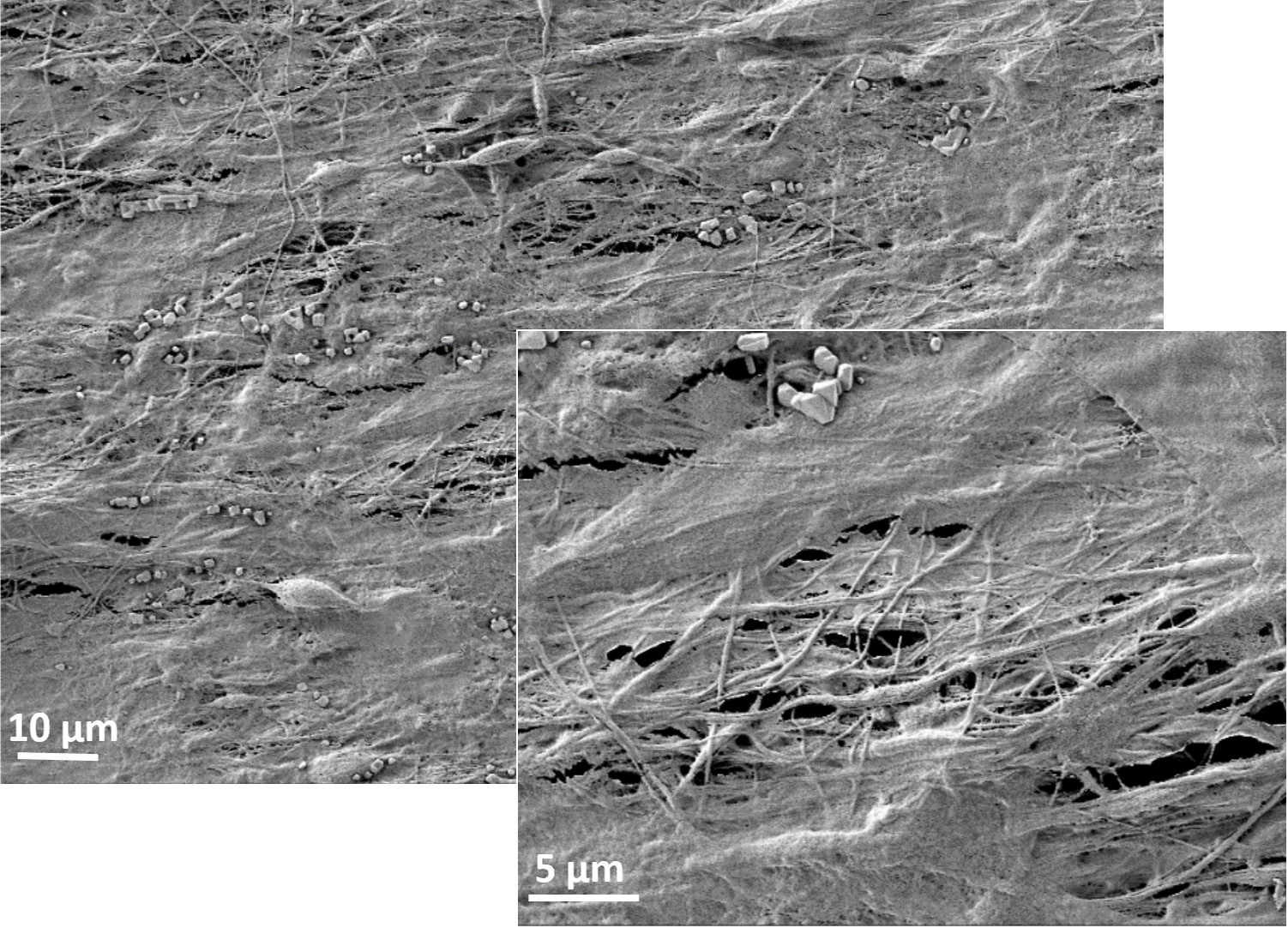

Supplement: Supplementary file 3 — Additional file 3 Scanning electron microscopy (SEM) of Mb and ADSC co-cultures on PCL-collagen I-PEO-nanofibers after 4 weeks of myogenic differentiation in serum-free medium + 25 ng/ml GDF11 [file 12860_2023_478_MOESM3_ESM.tif]
